# Social determinants of distress in South Asian men with long-term conditions: a qualitative study in primary care

**DOI:** 10.3399/BJGP.2024.0386

**Published:** 2025-05-07

**Authors:** Hassan Awan, Nadia Corp, Tom Kingstone, Carolyn A Chew-Graham

**Affiliations:** National Institute for Health and Care Research clinical lecturer; School of Medicine, Keele University, Keele; Research and Innovation Department, St George’s Hospital, Stafford.; School of Medicine, Keele University, Keele.

**Keywords:** distress, general practice, health inequality, long-term conditions, mental health, qualitative methods

## Abstract

**Background:**

People with long-term physical conditions are more likely to experience distress, depression, or anxiety. Physical–mental comorbidity is associated with lower quality of life, poorer clinical outcomes, and increased mortality than physical conditions alone. People of South Asian origin are the largest minority group in the UK, and more likely to have long-term conditions (LTCs) such as diabetes and heart disease.

**Aim:**

To explore how men of South Asian origin with LTCs understand and experience emotional distress as well as the experiences of GPs supporting them.

**Design and setting:**

A UK qualitative study interviewing South Asian men with diabetes or coronary heart disease, and GPs working at practices with higher proportions than average in the UK of people of South Asian origin.

**Method:**

Online semi-structured interviews with South Asian men and GPs were undertaken. Data were analysed via reflexive thematic analysis. Topic guides were modified iteratively as data collection and analysis progressed. An ethnically appropriate patient advisory group of South Asian men was involved in all stages of the research.

**Results:**

Seventeen South Asian men with LTCs and 18 GPs were interviewed. Participants described contextualising distress, including LTCs influencing distress and the intersections of social determinants of distress including ethnicity, poverty, and perceptions of prejudice. Participants understood distress as different from depression with the need to negotiate multiple identities as well as integrative paradigms of health.

**Conclusion:**

This study highlights the influence of social determinants of distress in South Asian men with LTCs. It provides an insight into how primary care has the potential to address health inequalities by considering the intersection of these factors.

## Introduction

People with long-term physical conditions are more likely to develop mental health problems such as depression and anxiety.^1^–^3^ People with physical–mental multimorbidity have poorer outcomes for quality of life and mortality outcomes.^4^–^6^ Deprivation is much more common in some ethnic minority communities in the UK; the highest rates of poverty are in people of Bangladeshi and Pakistani ethnicity at 50% and 46%, respectively, in comparison with 20% of people of White ethnicity.^7^ People from more deprived areas develop multiple long-term conditions (LTCs) 10–15 years earlier than people from affluent areas, in particular physical–mental comorbidity.^8^

People from ethnic minorities in the UK are under-represented in research, which may make health research less generalisable to this patient group and, inadvertently, maintain and/or widen health inequalities.^9^–^11^ People from ethnic minority groups may not recognise mental health problems yet have a higher prevalence of mental health problems, are less likely to seek help from services, and face discrimination as well as treatment inequalities, such as being more likely to be prescribed antidepressants and not referred for ‘talking therapies’.^12^–^14^ Stigma related to cultural understandings of health can be a barrier to seeking help and add complexity to consultations.^15^,^16^ Cultural trauma, whereby an oppressive dominant group assault another group’s culture, is a neglected but potential contributor towards health inequalities by reducing access to healthcare services.^17^

South Asian people (people of origin of India, Pakistan, Bangladesh, Afghanistan, Sri Lanka, Maldives, Nepal, Bhutan, and Indian Ocean Islands) are the largest minority group in the UK.^18^ Perspectives within South Asia of health and wellbeing incorporate physical, mental, and spiritual dimensions of health, emphasising traditional practices as well as family and community wellbeing.^19^ People of South Asian origin are more likely to have LTCs such as diabetes^20^ and heart disease.^21^ Diabetes and heart disease can be considered as exemplars (typical examples) of LTCs that share multiple similarities in terms of risk factors, symptoms, disease progress, and lifestyle changes, and have been described to have a ‘common soil’.^22^

**How this fits in**

Little is known about the understanding and experiences of emotional distress among South Asian men with long-term conditions (LTCs). This research explores important social factors that shape experiences of distress within this population, such as ethnicity, poverty, and perceptions of prejudice within health care. This research highlights the importance of the social determinants of distress in South Asian men with LTCs and how they can be considered in the primary care consultation.

Emotional distress includes upset and negative emotions that do not fit diagnostic criteria for mental illness.^23^ This term may be more acceptable to people of South Asian origin with LTCs who use non-medical terminology to describe emotional crises as opposed to mental health diagnoses such as anxiety and depression.^15^ Numerous studies have explored the mental health of South Asian women;^24^–^30^ however, a gap in knowledge exists to inform our understanding of experiences of distress among South Asian men with LTCs and the provision of care in general practice.

The qualitative study presented here asked the following questions:

- How do men of South Asian origin with LTCs understand and experience emotional distress?
- What are the experiences of GPs supporting men of South Asian origin presenting with distress?

## Method

This was a qualitative study that used semi-structured interviews to explore perspectives and understandings through the interpretation of experiences of men of South Asian origin and GPs informed by critical realism.^31^

### Setting and participants

The study took place in three regions of England. Men of South Asian origin aged ≥18 years with self-reported diabetes and/or coronary heart disease and GPs working in a general practice with a higher UK density of South Asian patients, either as a partner or salaried doctor, were recruited.

### Sampling and recruitment

Sampling of South Asian men with LTCs was purposive to support diversity. Recruitment took place via community channels, such as community and faith centres and shops frequented by South Asian communities, social media (Twitter/X and Facebook), and local radio. General practices from catchment areas with higher South Asian population densities of people of South Asian origin in Staffordshire, Manchester, and Lancashire were identified using the Department of Health and Social Care’s SHAPE atlas tool (https://shapeatlas.net). Practice managers were emailed and asked to distribute study information to GPs within their practice. This information included a research poster and participant information leaflet explaining the purpose of the study.

Snowball sampling,^32^ in which participants were asked to encourage other potentially eligible participants who may be willing to be interviewed, was used as a supplementary means to distribute information to South Asian men and GPs.

Participant information leaflets and consent packs were sent via email to potential participants before interview. Individuals who agreed to participate were invited to respond by telephone or email to arrange an interview.

### Data collection

One author (a male academic GP of South Asian background trained in qualitative research methods) conducted all interviews between November 2020 and October 2021. Interviews were conducted in English, although participants sometimes used terms from their native languages, which were understood by the interviewer.

Semi-structured interviews took place online and were one-to-one (interviewer: participant). Separate topic guides, one for South Asian men with LTCs and one for GPs, were developed from the literature and in discussion with the research team and patient advisory group (PAG). Topic guides were iteratively revised as informed by concurrent analysis.

Interviews were digitally recorded with consent, transcribed verbatim by a transcription company, and anonymised.

Data collection stopped after inductive data saturation was achieved, when the team was confident that the themes were sufficiently well-established and coherent.^33^ Reporting of this study concords with the Consolidated criteria for reporting qualitative research (COREQ) and the template has been completed.^34^

### Data analysis

Reflexive thematic analysis^35^ was used to analyse data. A constant comparative approach was also used to inform comparison within and across datasets from South Asian men and GPs; this technique has been conducted in studies with participants from different groups.^36^ Transcripts of audio-recordings were initially coded by one author using NVivo (version 12) software analysis. Codes were developed from quotes and evidenced accordingly; codes were then grouped into categories that were used to conceptualise themes. Negative case analysis was sought out and discussed.

### Patient and public involvement and engagement

A PAG of five South Asian men met five times during the course of the research and were involved in all stages, following National Standards for Public Involvement.^37^ The PAG contributed to the development of the research question, reviewed all public-facing documents to make them more culturally relevant, and supported recruitment, suggesting a poster in shops frequented by people of South Asian origin, and which shops specifically and in which areas. Members also suggested community and faith centres of different backgrounds and reaching out to local media that had a South Asian following. The PAG informed analysis and development of themes, and dissemination priorities. The PAG co-produced an animation video of key messages from the research (https://www.youtube.com/watch?v=pSuTAf_JU1I).

### Research quality

Reflexive field notes were taken during and after the interviews to support confirmability by ensuring awareness of personal feelings and thoughts, and how these could potentially influence the research and how they could be minimised. Triangulation of sources took place via comparison of interviews from GPs and people of South Asian origin.^38^ Triangulation through multiple analysis took place via different members of the research team and the PAG. The research team consisted of two academic GPs, one of whom was of South Asian background, an evidence synthesis specialist, and a social scientist. Credibility was maintained with monthly study meetings; researchers reflected on their professional and personal backgrounds and how these influenced their perspectives. Interviewing South Asian men and GPs from three different geographical areas in England makes the research potentially transferable to different geographical settings.

## Results

Seventeen South Asian men with LTCs and 18 GPs were interviewed, with most interviews lasting approximately an hour. Tables 1 and 2 describe the participant demographics for the South Asian men with LTCs and GPs, respectively.

**Table 1:** Characteristics of South Asian participants with LTCs

Participant ID	Self-identified ethnicity	Age, years	Index LTC	Comorbidities	Educational attainment	Occupation	Religion	Years in the UK	Generational status^a^
**SA01**	Pakistani	60	Diabetes type 2	None reported	College	Taxi driver	Islam	42	First
**SA02**	Pakistani	54	Diabetes type 2, coronary heart disease	None reported	College	IT consultant	Islam	51	First
**SA03**	Indian	62	Diabetes type 2	None reported	University	Operations manager	Islam	55	First
**SA04**	Indian	67	Diabetes type 2	None reported	No schooling	Retired factory worker	Islam	22	First
**SA05**	Pakistani	60	Coronary heart disease	Hypercholesterolaemia, prediabetes	University	IT consultant	Islam	50	First
**SA06**	Pakistani	39	Diabetes type 2	None reported	University	Unemployed	Islam	Born in UK	Second
**SA07**	Bangladeshi	42	Diabetes type 1	Hypercholesterolaemia, hypertension	College	Security worker	Islam	40	Second
**SA08**	Pakistani	57	Diabetes type 2, coronary heart disease	Hypercholesterolaemia, hypertension	Secondary school	Factory worker	Islam	45	First
**SA09**	Bangladeshi	33	Diabetes type 2	Asthma	University	Teacher	Islam	32	Second
**SA10**	Bangladeshi	37	Diabetes type 2	Paranoid schizophrenia	University	Unemployed	Islam	26	First
**SA11**	Pakistani	83	Diabetes type 2, coronary heart disease	Osteoarthritis	Secondary school	Retired travel agent	Islam	60	First
**SA12**	Pakistani	50	Diabetes type 2, heart disease	None reported	University	Post office and taxi driver	Islam	25	First
**SA13**	Pakistani	66	Diabetes type 2, heart disease	None reported	Secondary school	Businessman	Islam	52	First
**SA14**	Pakistani	62	Diabetes type 2	Partially blind	College	Driver on sick leave	Islam	35	First
**SA15**	Indian	63	Diabetes type 2	Dystonia	Partial secondary school	Post office	Hinduism	22	First
**SA16**	Pakistani	62	Diabetes type 2	Hypercholesterolaemia	Partial secondary school	Shopkeeper	Islam	31	First
**SA17**	Bangladeshi	30	Diabetes type 1	None reported	College	Underwriter	Islam	Born in UK	Second

a Two participants came to the UK very young (1 and 2 years old) and hence consider themselves second generation.

LTC = long-term condition.

**Table 2:** Characteristics of GP participants

Participant ID	Age, years	Gender	Self-identified ethnicity	South Asian languages spoken	Clinical sessions worked, *n*	Years of experience as a GP	Roles
**GP01**	64	Male	Indian	Gujarati	3	32	Partner; academia
**GP02**	40	Male	Pakistani	Punjabi, Urdu	3	11	Partner; leadership
**GP03**	55	Male	English	Nil	4	29	Salaried; medical education
**GP04**	39	Male	Pakistani	Urdu, Punjabi	6	10	Partner; medical education
**GP05**	50	Male	Pakistani	Urdu	9	20	Partner; leadership
**GP06**	38	Male	Indian	Gujarati	6.5	11	Partner; medical education
**GP07**	43	Male	Pakistani	Urdu, Pashto	6	8	Salaried; other clinical roles, medical education
**GP08**	33	Male	Pakistani	Urdu, Punjabi	6	4	Partner; medical education
**GP09**	32	Female	English	Nil	4	2.5	Salaried; academia, medical education
**GP10**	44	Male	Bangladeshi	Bengali	3.5	16	Partner; leadership, medical education
**GP11**	38	Female	Indian	Nil	6.5	6.5	Salaried
**GP12**	42	Male	Pakistani	Urdu, Punjabi, Hindko	8	5	Salaried (and locum); medical education
**GP13**	30	Female	Indian	Tamil	10	1	Partner
**GP14**	33	Male	Indian	Hindi	2	4.5	Salaried; other clinical roles
**GP15**	49	Male	Pakistani	Urdu	7.5	18	Salaried; other clinical roles
**GP16**	45	Male	Bangladeshi	Bengali	9	16	Partner; leadership
**GP17**	49	Female	Pakistani	Urdu	6	7	Salaried; other clinical roles
**GP18**	31	Male	Bangladeshi	Bengali	6	3	Salaried; leadership

Two main themes were developed: contextualising distress and conceptualising distress. Contextualising distress embodies the circumstances within which distress was experienced, which LTCs influence distress, and intersections of the social determinants. Conceptualising distress, that is, the formalisation of the understanding of distress, was understood in a variety of ways by men of South Asian origin and GPs, emphasising de-medicalising distress, negotiating multiple identities, and integrative paradigms of health. Themes are supported by illustrative quotes whereby names have been replaced with codes.

### Contextualising distress

#### ‘Betrayed by my body’. LTCs influencing distress

Experiences of being diagnosed with an LTC were a source of distress, being described by the South Asian participants with LTCs as stressful and shocking, as well as feeling let down by their bodies and anxious about future events. This was more pronounced in diagnoses of heart disease than diabetes:

*‘The diabetes at first, it was very depressing, that I felt my body had let me down. Obviously, from being quite active, bouncing about and football and whatever, whenever I could. I think I was forty-nine or something like that at the time. I felt a bit betrayed by my body. I know it’s silly. But then I got used to it and I find that it didn’t really affect me that much. I was okay, pretty much, within myself after a couple of months. And then with this heart attack now, it’s very depressing. Again, because now, I’m always worried about the next event.’* (South Asian [SA]02, Pakistani origin, aged 54 years, diabetes type 2 and coronary heart disease)

GPs described the challenges of supporting men of South Asian origin managing the burden of their LTC, and individuals tiring from this burden leading to reduced concordance and poorer control of their LTCs. A lack of engagement made it challenging to work with patients to make shared management plans:

*‘They might present to me because the nurse has said she doesn’t know what to do with them. Their diabetes is getting worse and worse control. You look and you realise that they haven’t been taking their tablets. Their compliance has been really low. When you challenge them on that they deny it completely and go, “oh no doctor I take my tablets all the time” … It’s because they just lost interest in looking after themselves.’* (GP05, GP partner, 20 years’ experience)

South Asian participants with LTCs described frustration managing their LTCs owing to the multiple demands of treatment such as dietary and lifestyle changes as well as medication. They described how this could lead to chronic stress that at times tipped into distress and disengagement from managing their health:

*‘Sometimes you almost erupt. We’re trying to sort out dietary needs and trying to do everything right concerning diabetes. When a diabetes nurse speaks to you, they tell you, you have to do this, you have to exercise, you must do this and you must do that. Certain things in your life can happen where you just think, forget this, I’ve had enough. How much longer am I going to put up with all this, or do I have to do this all my life? … And then there have been times where I have thought I’ve had enough of this or I can’t do this any more.’* (SA07, Bangladeshi origin, aged 42 years, diabetes type 1)

#### Intersections of social determinants contributing to distress

A number of social factors were described as intersecting to contribute to distress in South Asian participants living with an LTC, namely financial stress, family difficulties, and prejudice.

Financial pressures were described as more stressful than physical health problems, owing to the constant pressure of poverty affecting all aspects of a person’s life as well as directly affecting health:

*‘Financial thing is the main issue. If you don’t have the money, without money you can’t live. They say that health is more important, I always say money is more important than health, because if you don’t have the money, your health will go down anyway. You can’t eat the proper thing, you can’t pay the bill, your stress will come, you are short of everything.’* (SA13, Pakistani origin, aged 66 years, diabetes type 2 and coronary heart disease)

Of note, South Asian participants with LTCs described health as secondary to financial pressures, and did not describe health as a priority in itself. GPs explained how some South Asian men with LTCs did not seem able to prioritise their health because of competing problems, such as finances and poor housing, leading to presenting to primary care as a result of socioeconomic factors:

*‘Sadly, many present, and the triggers to their presentation tend to be, again, socioeconomic reasons. So, they’ve got financial issues, employment issues, problems within their relationships. And, sadly, lots of addiction problems … And the thread that links all of this across communities, beyond South Asian communities, I think, is deprivation.’* (GP02, GP partner, 11 years’ experience)

Family challenges were described as a great source of distress. Some South Asian participants with LTCs described a tension related to acculturation, struggling between the expectation they had of their children based on the South Asian culture they were brought up in, in contrast to the more British culture their children grow up in and act on. This tension was amplified in multigenerational households, more prevalent among people of South Asian origin from low socioeconomic classes (based on occupation), with family members being around each other often yet having different values:

*‘A lot of tension is tension with the family. Ninety-nine per cent of problems of tension is with family. It’s a family killer, these problems because we’re living with our families so much.’* (SA02, Pakistani origin, aged 54 years, diabetes type 2 and coronary heart disease)

Prejudice was described by South Asian participants with LTCs as a significant contributor to distress, such as the perceived biased nature of media coverage of people of South Asian origin and government policies unfairly targeting people of South Asian origin such as imposing a lockdown the night before an important South Asian festival while easing restrictions for Christmas:

*‘The fact that they announced the lockdown the night before Eid al-Fitr was extremely frustrating. And the fact that now, they’re pulling out all the stops, and they couldn’t care less about any increase in the infection rate for Christmas, it’s infuriating.’* (SA02, Pakistani origin, aged 54 years, diabetes type 2 and coronary heart disease)

Suffering across the world was also described as a source of distress, owing to perceptions of prejudice on a global level with loss of life considered insignificant based on where someone was from. When a South Asian participant described international injustices as a source of distress, the interviewer asked whether world politics makes him distressed, to which he replied:

*‘I’m sorry. It isn’t politics. No. It’s frustrating, people, if they say, oh forget all the things. They are human. How could it be politics? And then the Palestinian people, they are taking them and killing them. How can it be politics?’* (SA01, Pakistani origin, aged 60 years, diabetes type 2)

### Conceptualising distress

#### ‘A universal human experience’. De-medicalising distress

GPs described how emotional distress is a non-medical and normal human experience but, if left unchecked or untreated and develops beyond the capability a person is able to manage, this can lead to more severe problems:

*‘Emotional distress, I think, is a universal human experience, it’s a normal human experience. And I think that clearly there are different levels. There’s the day-to-day normal and then it becomes gradually more and more difficult to deal with, to survive. And eventually, at some point a person gets to their threshold where they think that it’s appropriate to consult their doctor or their healthcare professional.’* (GP03, salaried GP, 29 years’ experience)

South Asian participants with LTCs similarly differentiated distress from medical conditions such as depression. This was based on severity of symptoms, which had a window of treatment before escalating into a mental health disorder:

*‘It depends on how severe it is. I don’t know. Emotional distress could change to mental illness quite rapidly, and you could feel, I don’t know, trapped. You can’t do much, or you don’t have much of a challenge in your life.’* (SA17, Bangladeshi origin, aged 30 years, diabetes type 1)

GPs expressed concerns of an overly biomedical approach in which distress is medicalised by clinicians, patients, and society in general. This was felt to negatively have an impact on patients’ lives by making them feel they had a medical disorder and developing an external locus of control as someone ill and helpless, as opposed to feeling empowered to ask for help and be supported:

*‘The more we medicalise matters of emotional wellbeing, the more further away we get from a point of success for patients.’* (GP02, GP partner, 11 years’ experience)

South Asian men with LTCs and GPs described a conceptual need to de-medicalise distress. Negotiating multiple identities, of both participants with LTCs and GPs, will now be explored.

#### ‘Men don’t cry.’ Negotiating multiple identities

South Asian participants with LTCs described negotiating multiple identities based on ethnicity, living with LTCs, and their culture. From a GP perspective, culture was felt to be intertwined with mental health:

*‘I’ve always felt that psychiatry, mental health, along with everything else, it is culturally driven, as well as medically driven.’* (GP01, GP partner, 32 years’ experience)

Men of South Asian origin and GPs felt that, within South Asian culture, emotional health was viewed as less important than physical health. This ranged across different South Asian backgrounds, and was more prominent in first-generation participants:

*‘I think in the Indian subcontinent and our culture, we just see physical as being the most important, not emotional. Emotional, you should be able to manage yourself.’* (SA09, Bangladeshi origin, aged 33 years, diabetes type 2)

Part of South Asian culture regarding men was a concept of being breadwinners and not being allowed to show signs of weakness. This was more prominent in South Asian people who were first-generation, who would make all efforts to fulfil this role, and become increasingly distressed if unable to do so:

*‘Men don’t cry, and men should be strong, and they should look after their sisters and they should look after their wives and so forth. That’s the kind of narrative a lot of the middle to elderly* [South Asian] *population has grown up with.’* (GP16, GP partner, 16 years’ experience)

As well as complexities in South Asian men with LTCs negotiating multiple identities, GPs described professional identity as a source of tension. This included when abiding by their own personal culture and beliefs while supporting South Asian patients with often similar beliefs, yet feeling a necessity to keep this out of the consultation:

*‘We have to remain apolitical and areligious within our consultations themselves, and it’s a very fine balance. And that’s another challenge for us. That, whereas this person might have very strong religious beliefs, and I personally might also have very strong religious beliefs. We’ve got to keep that out and keep it as neutral as possible.’* (GP05, GP partner, 20 years’ experience)

#### ‘I believe in black magic.’ Integrative paradigms of health

Integrative paradigms of health, which synthesise conventional and complementary medicine, were described by some South Asian participants with LTCs. These included a greater trust in traditional forms of medicine more commonly used within South Asian countries such as via Hakeems (traditional doctors, from the root word meaning ‘wisdom’). People of South Asian origin described frustration with UK medicine being focused on multiple tests as opposed to traditional doctors who they described would look for causes of illness and offer holistic and natural cures:

*‘In the old days, I remember*, [in] *all villages, there was good Hakeems* [traditional doctors] [who] *always helped you. And these days, the doctors say have this test and that test.’* (SA11, Pakistani origin, aged 83 years, diabetes type 2 and coronary heart disease)

Other paradigms of health included concepts of black magic and supernatural beings. These were described as potential causes of mental as well as physical illness, with participants querying if their experiences of distress were related to these:

*‘I believe in black magic, I believe in Jinn* [supernatural beings], *I believe in Hasad* [envy that affects others], *I believe in the evil eye. Me, personally, sometimes I do think, am I possessed? Is this what causes my illness?’* (SA10, Bangladeshi origin, aged 37 years, diabetes type 2)

Black magic was ingrained within South Asian culture, and was perceived as a cause of difficulties across the sphere of a person’s life, including causing social and relationship problems, with families encouraging cultural treatments:

*‘So, I think for about a year or two, when I became very unwell, that’s what the discussion was in the family and the community, that someone had done black magic, which wasn’t the case. It was mental ill health. I was suffering from distress over the fact that my relationship didn’t turn out to be as it should have been. So, I think that’s what it was. But a lot of the community, the first-generation community of South Asians, they have that opinion, a lot of them, that it’s black magic. We need to take you to a religious leader. We need to give you this Taweez, this ribbon for you to wear. So, that’s really it. There is still that going on, because mental ill health is largely misunderstood in the South Asian community.’* (SA06, Pakistani origin, aged 39 years, diabetes type 2)

Concepts of black magic were almost never discussed by GPs. This may be because South Asian men with LTCs thought they would be perceived as mentally unwell. If mentioned by GPs, they were attributed to severe mental illness whereby other services would be involved. When directly asked about patients describing black magic a GP replied:

*‘In the twenty years, I’ve probably had that about two or three times. Tends to be more with those at the severe end of mental illness. The ones who are psychotic and suffering with bipolar disorder and those sorts, at the severe end. Those patients are well known to the mental health services. They’ve had counselling and support, and continue to do so. I don’t really get involved in that, those patients as much.’* (GP05, GP partner, 20 years’ experience)

**Figure 1:**
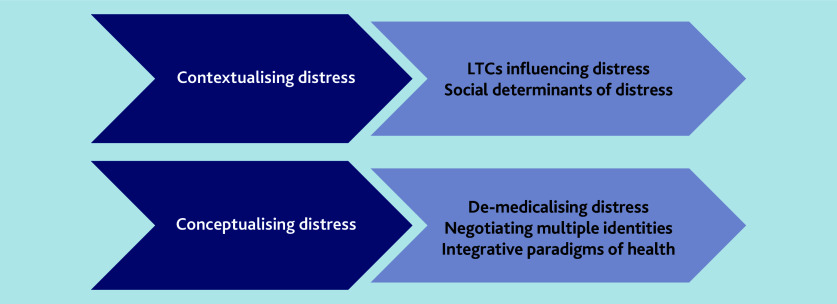
Summary of overall themes. LTC = long-term condition.

The themes are summarised in Figure 1.

## Discussion

### Summary

This study explored how men of South Asian origin with LTCs experience and understand emotional distress from the perspectives of both people of South Asian origin and GPs. South Asian participants with LTCs and GPs described contextualising distress in terms of factors that contributed to their distress; this included living with LTCs and intersections with key social determinants such as family stressors, poverty, and perceptions of prejudice. Conceptualising distress — how participants interpreted and made sense of their distress — highlighted conceptual differences between emotional distress as a universal experience and clinical depression, challenges in negotiating multiple identities linked to faith, culture, masculinity, and having an LTC, and integrative paradigms of health including traditional doctors and concepts of black magic. Both the contextualisation and conceptualisation of distress are needed to fully understand experiences of distress in South Asian men.

### Strengths and limitations

This study provides new insights into emotional distress in South Asian men with LTCs. An ethnically appropriate PAG of South Asian men acting as partners in the research, as well as a South Asian ‘insider researcher’, were crucial to making the research relevant and facilitated participants to be more open to talk about issues around culture and mental health such as black magic; a previous study has shown that lack of knowledge of South Asian culture hindered communication owing to having different worldviews and understandings.^39^

Community sampling took place that facilitated listening to the voice of people from the South Asian community who may not otherwise be heard. The co-creation of an animation video with the PAG summarising the key messages from the research provided a platform for dissemination for people who may be neglected by other forms of dissemination.

Limitations include interviews being all remote because of restrictions associated with the COVID-19 pandemic. Virtual meetings are potentially more accessible than in-person interviews, are preferred by interviewees, and have similar interview lengths, subjective interview ratings, and substantive coding, although they are less word-dense.^40^ Although South Asian participants with LTCs came from the three main South Asian countries, the majority were of Muslim faith.

### Comparison with existing literature

The theme of contextualising distress identifies key social factors contributing to distress in South Asian men, aligning with literature on the social determinants of mental health.^41^ This literature describes a reciprocal relationship where mental health problems and social determinants influence each other.^42^ The social determinants of distress are more relevant for people of South Asian origin given their resistance to the medicalisation of distress observed in our study. People from ethnic minority communities are more likely to experience socioeconomic deprivation, related to a wider social context influenced by racism, including in housing, employment, and the criminal justice system.^43^ GPs and the participants of South Asian origin highlighted how social determinants such as ethnicity, LTCs, financial pressures, and prejudice influence distress.

A Race Equality Foundation report noted an increased prevalence of mental illness among ethnic minority communities, particularly Pakistani men, alongside reduced access to mental health services, assessment and treatment inequalities, and discrimination during recovery.^14^ The allostatic load of the cumulative burden of chronic stress and life events, described by participants both in terms of social determinants as well as coping with their LTC, is known to lead to poorer health outcomes.^44^ A systematic review on stigma associated with common mental health disorders found a link to poverty and discrimination from policies and institutions.^44^ Our study enhances the understanding of prejudice in health care and how broader policies, such as those around COVID-19, directly influence distress in people of South Asian origin.^45^ This exemplifies intersectionality, where multiple social forces, identities, and ideologies intersect to create disadvantage. GPs and the participants of South Asian origin in our study described the various levels of intersectionality they encounter.

Reflecting on the intersectionality faced by people of South Asian origin can improve patient–clinician interactions by fostering a better understanding of multiple disadvantages and the institutional and structural forces leading to health disparities. It also prompts reflection on GPs’ positions of power and privilege, and how these, along with their biases, have an impact on consultations.^46^,^47^ This may include an impact on GPs who feel they are not able to speak about faith related to distress when the patient is keen for such a discussion. Addressing social determinants of health within primary care involves developing a practice culture that values health equity and employs a team-based approach.^48^ As Moscrop *et al* asks, *‘If social determinants of health are so important, shouldn’t we ask patients about them?’*^49^

The participants of South Asian origin and GPs in this study conceptualised distress as a distinct entity to depression and other mental health diagnoses. They described a continuum whereby if distress was prolonged and severe it could lead to depression. Research has shown that people who present to primary care can be differentiated to have distress or depression based on symptomatology.^50^ Cultural differences have been found with presentations of mental health problems, for example, South Asian people with LTCs have been shown to prefer non-medical terminology, such as tension, to medical terminology.^15^,^16^ A previous study found that some GPs felt distress and depression were on a continuum and distinction was not possible, whereas other GPs struggled to separately define the entities and others linked distress with the absence of biological symptoms.^51^ Men in general in the UK prefer to use non-clinical terminology when engaging with mental health services, similar to the views of South Asian men in this study.^52^ Differentiating distress from depression can aid appropriate diagnosis and hence suitable management of both distress and depression, potentially reducing inappropriate diagnosis of depression.^50^ There are cultural tensions with the term depression; an Asian population described no benefit of using the term depression above non-medical descriptions of unhappiness.^53^ De-medicalising distress provides a platform for South Asian conceptualisations of distress to be valued not only in the minds of patients but also authenticated and validated by clinicians in the consultation setting.

### Implications for practice

Box 1 summarises how the themes can support clinicians in incorporating the social determinants of distress to CARE (Cultural sensitivity, Ask, Reflect, Explore/engage) for South Asian men with LTCs. By adopting a person-centred approach during consultations with South Asian men with LTCs, one that negotiates gendered stereotypes and acknowledges sociocultural backgrounds, GPs could encourage more patients to disclose experiences of distress using their preferred terms and language, and, where needed, improve treatment planning, which may include discussing traditional forms of medicine. These measures, all of which will act to improve patient–doctor relationships, build trust, and protect and enhance the professional integrity of the clinician, are essential for quality health care.

Box 1 **Incorporating the social determinants of distress to CARE for South Asian men with LTCs**

**Table:** 

**Cultural sensitivity**	Be sensitive and open to different conceptualisations of distress, including the multiple identities that South Asian men with LTCs have to navigate, such as a breadwinner role that may perceive distress as a sign of weakness Consider integrative paradigms of health such as black magic causing distress
**Ask**	Ask about the social determinants that may influence distress, such as distress related to the LTC, financial pressures, family challenges, and experiences of prejudice
**Reflect**	Reflect on one’s own professional identity, including clinician power and privilege and biases, and how this influences the consultation, in a positive or negative way
**Explore/engage**	Explore distress using language the patient is comfortable with, which may be non-medical terminology to de-medicalise distress, such as tension, as opposed to diagnostic labelling such as depression and anxiety

LTC = long-term condition.
